# Ectopic expression of a small cell lung cancer transcription factor, INSM1 impairs alveologenesis in lung development

**DOI:** 10.1186/s12890-016-0215-3

**Published:** 2016-04-12

**Authors:** Chiachen Chen, Mary B. Breslin, Michael S. Lan

**Affiliations:** Research Institute for Children, Children’s Hospital, 200 Henry Clay Avenue, Research and Education Building, Room. 2211, New Orleans, LA 70118 USA; Departments of Pediatrics, Louisiana State University Health Sciences Center, New Orleans, LA 70112 USA; Departments of Genetics, Louisiana State University Health Sciences Center, New Orleans, LA 70112 USA

**Keywords:** INSM1, SCLC, CCSP, Alveologenesis, Lung development, Transgenic model, Cyclin D1

## Abstract

**Background:**

Insulinoma associated-1 (*INSM1*) gene is expressed exclusively in early embryonic neuroendocrine tissues, but has been found highly re-activated in most of the neuroendocrine tumors including small cell lung carcinoma.

**Methods:**

In order to elucidate the functional effects of INSM1 in normal lung development, we used a conditional lung-specific INSM1 transgenic mouse model. Transgenic (Tet-on system) CMV-INSM1 responder mice were bred with the lung-specific, club cell secretory protein (CCSP) promoter-rtTA activator mice to produce bi-transgenic progeny carrying both alleles, CCSP-rtTA and Tet-on-INSM1. Mice were fed with doxycycline containing food at the initial mating day to the postnatal day 21. Lung samples were collected at embryonic day 17.5, newborn, and postnatal day 21 for analyses.

**Results:**

Northern blot, RT-PCR, and immunohistochemical analyses revealed that doxycycline induced respiratory epithelium-specific INSM1 expression in bi-transgenic mice. Samples from postnatal day 21 mice revealed a larger lung size in the bi-transgenic mouse as compared to the single-transgenic or wild-type littermates. The histopathology results showed that the alveolar space in the bi-transgenic mice were 4 times larger than those in the single transgenic or wild-type littermates. In contrast, the size was not significantly different in the lungs collected at E17.5 or newborn among the bi-transgenic, single transgenic, or wild type mice. The respiratory epithelium with INSM1 ectopic expression suppressed cyclin D1 signal. Further in vitro studies revealed that the ectopic expression of INSM1 suppresses cyclin D1 expression and delays cell cycle progression.

**Conclusion:**

The current study suggests that CCSP promoter-driven INSM1 ectopic expression impairs normal lung development especially in postnatal alveologenesis.

## Background

The lung produces more than 40 cell types to fulfill the important functions in mucociliary clearance, gas exchange, metabolism, and endocrine activities. The major cell types in lung epithelium include ciliated cells, basal cells, type I and II pneumocytes, club cells, and neuroendocrine (NE) cells [1]. Although pulmonary neuroendocrine cells (PNECs) are the first specialized cell type within the bronchial epithelium with progenitors of NE nature, their presence in lung are relatively scarce. Usually, a single pulmonary NE cell is scattered in the respiratory epithelium. Clustered PNECs, also called neuro-epithelial bodies (NEBs) are commonly found at bronchio-alveolar duct junctions. The ontogeny of PNECs and their relationships to other lung cells during normal homeostasis, lung injury, and late stages of lung development are still unclear. In naphthalene-induced lung injury, most club cells were damaged by the drug whereas those located nearby NEBs survived and were capable of restoring the damaged lung epithelium. These results suggest that the potential function of PNECs is to maintain the stem cell niches required for club cell regeneration and injury repair [2, 3].

Insulinoma associated-1 (IA-1/INSM1) was originally cloned from a human insulinoma subtraction library [4]. It was mapped to chromosome 20p11.2 as an intronless gene that translates into a protein of 510 amino acids and a predicted molecular mass of 52,923 Da [5]. *INSM1* gene is expressed exclusively during early embryonic NE development, but has been found highly re-activated in NE tumors [6]. INSM1 is a sensitive marker for NE differentiation in human lung tumors. INSM1 mRNA was detected by Northern blot analysis in 97 % (30 of 31) of small cell lung cancer cells and 13 % (4 of 30) of non-small cell lung cancer cells with NE phenotype. In most of the lung cancer cells examined, INSM1 expression showed high concordance with the other specific NE markers, synatophysin, L-dopa decarboxylase, and chromogranin A [7, 8]. An aggressive type of NE tumor, small cell lung carcinoma (SCLC) accounts for approximately 10 to 15 % of all lung cancers. INSM1 can be detected at high levels in most of the SCLC cancer tissues [7].

In order to determine the effect of INSM1 on normal lung development, we generated a conditional lung-specific INSM1 transgenic mouse model. In this model, the ectopic expression of INSM1 was selectively induced in non-ciliated bronchial epithelial club cells. Transgenic Tet-on-INSM1 responder mice were bred with the lung-specific, club cell secretory protein (CCSP) promoter-rtTA activator mice to generate bi-transgenic progeny carrying both alleles, CCSP-rtTA and Tet-on-INSM1. In this bi-transgenic model, INSM1 expression is induced by doxycycline (Dox) bound to rtTA, which in turn activates the Tet-on-CMV promoter, activating transcription of the *INSM1* gene. Our model provides a tool to elucidate the effect of INSM1 on PNECs. In the present study, we found that ectopic expression of INSM1 in bronchiolar epithelial cells impairs alveolarization resulting in alveolar space enlargement at the end stage of lung development. Ectopic expression of INSM1 inhibits cyclin D1 expression in the INSM1/rtTA bi-transgenic mouse bronchiolar epithelium and delays cell cycle progression. Our results suggest that INSM1 not only plays a role in alveolar septation, but also indicates that INSM1 might have profound effects on PNECs proliferation and club cell regeneration when pulmonary epithelium was damaged.

## Methods

### Animals and genotyping

For (tetO)_7_CMV-INSM1 mice, a human INSM1 full-length cDNA (2.8 kb) was sub-cloned into a pBI-EGFP Tet vector containing the CMV promoter and tetracycline response element. The transgenic animal model was generated from Gene Targeting & Transgenic Facility, University of Connecticut Health Center (Farmington, CT). Two lines of transgenic mice bearing (tetO)_7_CMV-INSM1 transgene were generated. The lung-specific Dox inducible CCSP-rtTA^-/tg^ transgenic line was obtained from Jackson laboratory. Bi-transgenic mice, named *INSM1/rtTA*, were generated by crossing (tetO)_7_-CMV-INSM1 and CCSP-rtTA^-/tg^. Wild type littermates lacking either rtTA or INSM1 allele were used as control. Transgenic mice were genotyped by PCR using genomic DNA from tail of fetal or postnatal mice. PCR primers for transgenes were: for CCSP-rtTA, forward primer 5’-ACTGCCCATTGCCCAAACAC-3’; reverse primer: 5’-AAAATCTTGCCAGCTTTCCCC-3’, for (tetO)_7_CMV-INSM1, forward primer: 5’-CCTTGTACAACCGACAGCTC-3’, reverse primer: 5’-GAGTGAGCTGATACCGCTCG-3’. The PCR amplification was performed as follow: denatured at 95 °C for 3 mins followed by 35 cycles of amplification at 95 °C for 30s, 58 °C for 30s and extension at 72 °C for 30s. Animals were maintained in a pathogen-free vivarium in filtered cages according to the protocol approved by Institutional Animal Care and Use Committee from the Research Institute for Children, Children’s Hospital in New Orleans. All mice were maintained in C57BL/6 background. Dams bearing double transgenes were fed with Dox food (200 mg/kg; Bio Serv co., Frenchtown, NJ) for various time spans.

### Cell Culture

A human normal bronchial epithelial cell line, BEAS-2B was obtained from American Type Culture Collection. The cells were cultured in Dulbecco’s modified Eagle’s medium (DMEM, high-glucose) with 10 % fetal calf serum (Atlanta Biological Inc., Norcross, Georgia), 1X Pen/Strep (10,000 IU penicillin and 10,000 ug/ml streptomycin) (Mediatech, Inc., Manassas, VA.) in a 5 % CO_2_ incubator at 37 °C.

### MTS assay

The 3-(4,5-dimethyl-2-yl)-5-(3-carboxymethoxyphenyl)-2-(4-sulfophenyl)-2H-tetrazolium, inner salt, MTS proliferation assay was carried out according to the manufacturer’s protocol (Promega Co., Madison, WI). The cells were seeded and infected with Ad-LacZ or Ad-INSM1 virus in a serum-free medium for 24 h and then cultured in Dulbecco’s modified Eagle’s medium (DMEM, high-glucose) with 10 % fetal calf serum. After culturing for 48 or 60 h, CellTiter 96® AQ_ueous_ One Solution Reagents were added into the culture medium and then incubated at 5 % CO_2_, 37 °C for 4 h. The amount of soluble formazan produced by cellular reduction of MTS was measured for absorbance at 490 nm using a microplate spectrophotometer to calculate the cell viability.

### RNA isolation and Northern blot analyses

Lung RNAs were extracted using TRIzol reagent (Life Technologies, San Francisco, CA) following the manufacturer’s instruction. RNA was treated with 2 units of DNase (Promega Co., Madison, WI) at 37 °C for 30 min to remove residual genomic DNA. Total lung RNA was used as template to synthesize cDNA by High Capacity RNA-to-cDNA™ Kit (Life Technologies, San Francisco, CA) following the manufacturer’s protocol. RNA was reverse transcribed and analyzed by PCR and/or real-time PCR for the expression of INSM1 and cyclin D1. The relative RNA concentration of the target gene was normalized to the concentration of the housekeeping gene, GAPDH. Primers for INSM1: INSM1-440aa 5’-ACGGAATTCTGCCACCTGTGCCCAGTGTGCGGAGAG-3’, INSM-510aa 5’-CACCTCGAGCTAGCAGGCCGGGCGCACGGGCACCTGCAG-3’ Primers for cyclin D1, forward 5’-TGCCTACAGCCCTGTTACCT-3’ and reverse 5’-ACTTTGCAGGACAGATCCCG-3’. Total RNA (20 ug) was separated on 1 % agarose/formaldehyde gel. The gel was transferred to a nitrocellulose membrane for 3 h in 20X SSC and then UV cross-linked. The membrane was pre-hybridized in Express Hybe Solution (Clontech, Mountain View, CA) for 1 h followed by hybridization in the same solution with ^32^P-labeled INSM1 probe, washed, and exposed to autoradiography.

### Western blot analyses

Cell lysates were extracted with the lysis buffer (10 mM Tris-HCl pH 7.5, 150 mM NaCl, 10 % glycerol, 1 % Triton X-100, 1 mM DTT, 0.2 mM PMSF, 1 ug/ml aproptinin, 1 ug/ml leupeptin, 1 mM Na_3_VO_4_ and 1 mM NaF), separated on 10 % SDS-PAGE gel, and transferred onto the nitrocellulose membrane (Bio-Rad Laboratories, Inc., Hercules, CA). The membrane was blocked with 5 % BSA in TBST (20 mM Tris-HCl pH 7.6, 137 mM NaCl and 0.1 % Tween-20), probed with specific primary antibody at 4 °C overnight, and bound with HRP-conjugated secondary antibody (Bio-Rad) at room temperature for 1 h. The membrane was developed with a chemi-luminescence substrate (Bio-Rad Laboratories, Inc., Hercules, CA), and the blot was autographed onto a X-ray film (Fuji Photo Film Co., Japan).

### Histology and immunohistochemistry

Human small cell lung carcinoma tissue array (LC802b) was used in this study. Each specimen collected from any clinic was consented to by both hospital and individual. Discrete legal consent form was obtained and the rights to hold research uses for any purpose or further commercialized uses were waived (US Biomax, Rockville, MD). An IRB exemption (#8885) was obtained from Louisiana State University Health Sciences Center, New Orleans. Mouse tissue sections were prepared from fetal (E17.5), neonatal (newborn and postnatal days 7, PN7), and 3-week old mice (PN21) fixed with 4 % paraformaldehyde in phosphate-buffered saline (PBS) at 4 °C. Tissue sections were stained by hemotoxylin and eosin (H&E staining) for the histopathology study. For immunohistochemistry, sections were blocked with 5 % BSA in PBS and incubated with either anti-CCSP, synatophysin, or cyclin D1 antibody (Cell Signaling Technology, Beverly, MA) at 4 °C overnight. Then, the slides were incubated with secondary antibodies conjugated with HRP for 1 h at room temperature and developed using a diamino-benzidine (DAB) histochemistry kit (Life Technologies, San Francisco, CA). For immunohistochemical staining of INSM1, anti-INSM1 antibody (Santa Cruz biotechnology, Inc., CA) with a MACH 3 biotin free polymer detection kit was used on the human tissue array slide and Mouse-on-Mouse HRP-polymer bundle kit were used on mouse tissue sections (Biocare Medical, Concord, CA).

### Lung morphometric analysis

For preparing the mouse lung samples, we euthanized mice with overdosed ketamine/xylazine, open the chest cavity, removed the sternum and ribs to expose the heart. We inserted the butterfly needle into the right ventricle and nicked at the right atrium to build a direct route to pulmonary circulation for lung perfusion and fixation with cold PBS and formalin. After perfusion, we injected 2 ml of 10 % formalin into trachea to fix and inflate the lung. The dissected lungs were measured by weight. The preserved lung samples were subjected to histological analysis. Lung tissue sections were stained with hematoxylin and eosin. Sections were visually scanned for position-matching regions compared with controls. At least four representative lobes were selected from each animal. A Kodak MI image system was used to measure terminal air space area. We drew the outline of the alveoli and used software to measure the area size at 200X magnification. For each sample, we measured 4 fields and each field at least 20 alveoli. The air spaces were distinguished from tissue based on intensity and the number of pixels acquired for each air space when converted to square micrometers.

### Flow cytometry

The cells were seeded and infected with Ad-LacZ virus or Ad-INSM1 virus for 48 or 96 h. Cells were collected and fixed with cold 70 % ethanol at 4 °C overnight. The fixed cells were washed twice with PBS and then the cells were incubated in PI staining solution (PBS with 0.2 mg/mL DNase-free RNase, 0.1 % Triton X-100, and 1 mg/mL propidium iodine) at room temperature for 30 min before analysis on the flow cytometer.

### Statistical analysis

Values were corrected and expressed relative to an control group. All experiments were repeated three times. Results are presented as mean ± SD. Statistical analysis was performed using either the Student’s t-test when only two groups were in the experiment or by an one-way ANOVA comparison of multiple groups using the Tukey-Kramer test with differences at *p* value of less than 0.05 being considered significant.

## Results

### INSM1 is a sensitive small cell lung cancer marker

**Fig. 1:**
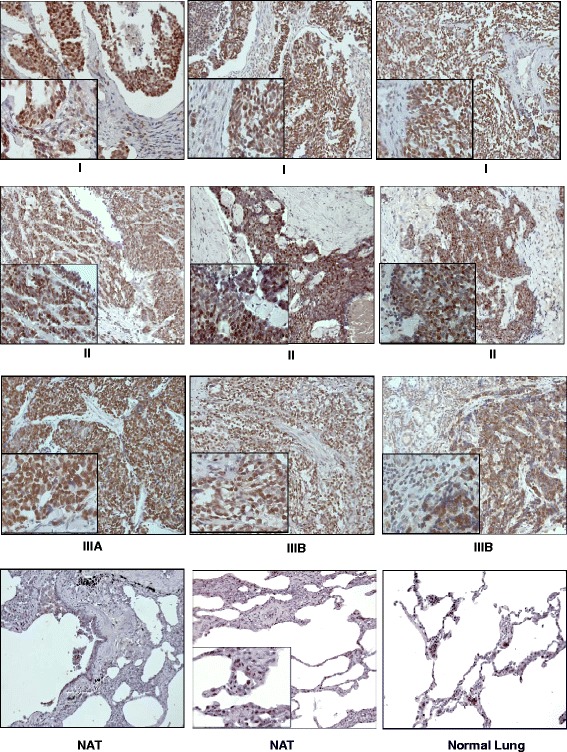
INSM1 staining of small cell lung cancer tissue array. Nine slides were selected from 35 cases of SCLC tissues (include clinical stages I, II, IIIA, and IIIB) and 3 normal lung tissues. Tissue array was immunostained with anti-INSM1 antibody. NAT: lung normal adjacent tissue. Original magnification is 200X and 400X (inset)

Small cell lung cancer tumors are derived from pulmonary NE cells (PNECs), therefore their antigenic profile coincides with that of NE cells. In this study, we used immunohistochemical staining to examine 35 cases of different clinical stages of small cell lung cancer and 5 normal lung tissues for INSM1 expression. All the small cell lung cancer tissues were strongly positive for INSM1. INSM1 signal was not detected on normal adjacent tissues from lung cancer patients or normal lung tissues (Fig. 1). The expression pattern of INSM1 in NE lung cancer is consistent with the previous Northern blot analysis that revealed INSM1 mRNA is highly expressed in nearly 100 % of small cell lung carcinomas (SCLC) cell lines but not in normal adult lung tissues [6, 7]. Here, we showed that the INSM1 protein is highly over-expressed in 35 SCLC tumor tissues confirming that INSM1 is a specific and sensitive NE marker of small cell lung cancer. However, the functional role of INSM1 in NE lung cancer or normal lung in PNEC development is still unclear.

### Ectopic expression of INSM1 in bi-transgenic animals

**Fig. 2:**
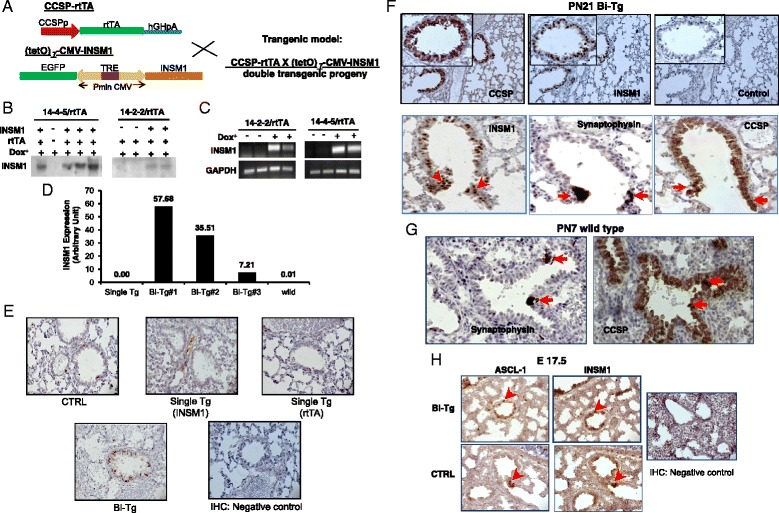
Generation of INSM1 transgenic model. **a** A 2.8 kb full length human INSM1 cDNA was cloned into a tetracycline operator expression cassette at pTet-on-Advanced expression vector. The expression cassette consists of seven tetracycline operator repeats (tetO), and a bi-directional CMV mimimal promoter for EGFP and INSM1 expression. The bi-transgenic mice (Bi-Tg) were generated by crossing (tetO)_7_-CMV-INSM1 and CCSP-rtTA transgenic mice controlled by 2.3 kb CCSP promoter-linked rtTA sequence and a human growth hormone poly(A) tail. In bi-transgenic mice (CCSP-rtTA x (tetO)_7_CMV-INSM1), rtTA is expressed in epithelial cells under the control of CCSP promoter. In the presence of doxycycline (Dox), rtTA binds to TRE element at (tetO)_7_CMV promoter and activates the expression of INSM1. **b** Bi-transgenic mice were fed with Dox food while mating. INSM1 mRNA were assessed at postnatal days 21 (PN21) by Northern blot analysis, **c** RT-PCR, and **d** real-time RT-PCR. INSM1 over-expression was detected in the lung tissues of bi-transgenic mice. No INSM1 expression was detected in single transgenic or wild type mice. Without Dox induction, no INSM1 expression in lung was detected. **e**, **f** INSM1 expression is detected in Bi-Tg lung and co-localized with CCSP and synaptophysin. No INSM1 signal was detected in wild type or single transgenic mice. **g** PNEC marker, synaptophysin-positive cells are CCSP-positive in wild type PN7 mouse lung tissue. **h** At E17.5 lung tissues, INSM1 expression is detected and co-localized with ASCL-1 in wild type and Bi-Tg. Results were represented from at least three mice

**Fig. 3:**
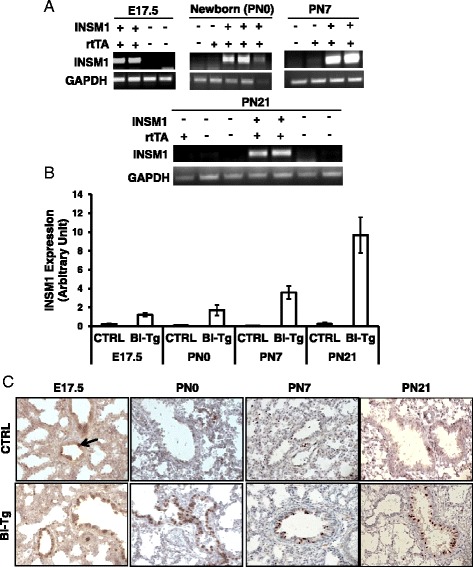
Expression of INSM1 in developing lung. **a** INSM1 transgene expression was detected by RT-PCR. **b** Relative quantities of INSM1 transcript was normalized with internal control, GAPDH. **c** Immunohistochemical staining for INSM1 protein. INSM1 transgene expression in Bi-Tg lung was readily detected in E17.5, newborn (PN0), postnatal day 7 (PN7), and PN 21. Endogenous INSM1 expression was detected in E17.5 (*arrow*) but scarcely in post-natal lung. Results were represented from at least three mice

In order to determine the effect of INSM1 in normal lung development, we used a conditional lung-specific INSM1 transgenic mouse model. Transgenic Tet-on-INSM1 responder mice were bred with the lung-specific, club cell secretory protein (CCSP) promoter-rtTA activator mice to generate bi-transgenic progeny carrying both alleles, CCSP-rtTA and Tet-on-INSM1 (Fig. 2a). In this bi-transgenic model, INSM1 expression is induced by binding the tetracycline analogue Dox to rtTA, which in turn activates the Tet-on-CMV promoter and the transcription of *INSM1* gene. Dox containing food was fed from the initial mating day to the weaning day (postnatal day 21, PN21) to ensure the full effect of INSM1 expression during lung development. Lung samples were collected at embryonic day (E) 17.5, newborn (PN0), and 3-week wean day (PN21). INSM1 was selectively expressed in a subset of respiratory epithelial cells, bronchial, and type II epithelial cells of lung tissues. The ectopic over-expression of INSM1 was spatially and temporally under the control of the lung specific CCSP-promoter and Dox. Two Tet-on-INSM1 transgenic lines (named 14-4-5 and 14-2-2) were generated and included in this study. Previous studies indicated that the CCSP-promoter directs rtTA transgene expression as early as post-conception day 14, E14 [9]. To ensure the fidelity of our transgenic system in regulating INSM1 expression, we treated the bi-transgenic (*INSM1/rtTA*), single transgenic (*INSM1* or *rtTA*), and wild type mice with Dox food from the beginning of conception day. At each time point, E17.5, newborn (PN0), postnatal day 7 (PN7), and postnatal day 21 (PN21), the lung tissues were collected and subjected to Northern blot analyses, RT-PCR, real-time PCR, and immunohitochemical staining for INSM1 expression (Fig. 2). The results revealed that Dox food induced respiratory epithelium-specific INSM1 over-expression in the bi-transgenic mice as compared to single transgene, or wild-type littermates (Fig. 2b and e). INSM1 over-expression was detected in both lines of bi-transgenic lung tissues under Dox induction. Without Dox induction there was no INSM1 signal in bi-transgenic lung tissues (Fig 2c). The INSM1 expression level varies in different bi-transgenic animals (Fig. 2d). Since the over-expression of INSM1 is under the control of CCSP-promoter, the immunohistochemical staining showed that INSM1 over-expression was co-localized with CCSP expressing cells (Fig 2f). We performed the immunohistochemical staining of transgenic mouse tissues with anti-CCSP, anti-INSM1, or anti-synaptophysin antibody using the consecutive tissue sections from PN21 Bi-Tg, and PN7 CTRL lungs (Fig. 2e, f, g). We observed that a few bronchiolar epithelial cells with synaptophysin signal are CCSP-positive on the CTRL mouse lung section (Fig. 2g). On PN21 Bi-Tg lung section, INSM1-positive cells are also CCSP positive (Fig. 2f). We detected ASCL-1 and INSM1 expression on the consecutive tissue sections from E17.5 wild type and Bi-Tg lungs (Fig. 2h). The ASCL-1 positive cells are INSM1-positive. Synaptophysin and ASCL-1 are well known NE cell markers and were used as PNEC markers. The bi-transgenic lung was subjected to a time course study (Fig. 3a). The INSM1 transcript is expressed in all four time points, from E17.5, PN0, PN7, to PN21. Among the lung tissues that we collected from both lines of animals, the expression of transgenic INSM1 was significantly increased with age at the RNA level (Fig. 3b). The immunohistochemical staining of INSM1 showed that the over-expression of INSM1 on bronchial epithelial cells of bi-transgenic mice is consistently positive from embryonic stage (E17.5) until PN21. However, INSM1 expression in normal lung is only scarcely detected in E17.5 fetal lung (Fig. 3c). Although over-expression of INSM1 mRNA was increased by age, the protein levels were not significantly increased in a time-dependent manner.

### INSM1 expression alters lung morphology

**Fig. 4:**
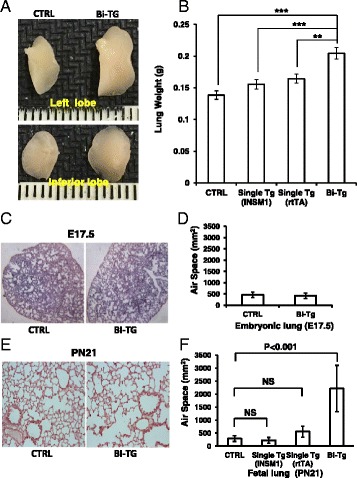
Lung morphological changes in bi-transgenic mice. **a** Lungs from left and inferior lobes were collected at PN21. **b** Lung size from *INSM1/rtTA* bi-transgenic mice (Bi-TG) in all lobes was compared to that of wild type control littermates, and single transgenic mice. The lung size was measured by weight scale after the lung was inflated with 10 % formalin via trachea. Bi-transgenic mice and the control littermates were treated with doxycycline from E0 to the time when lungs were sampling. Lung sections were stained with H&E on E17.5 (**c**, **d**) and PN21 (**e**, **f**). The original magnification is 200X. Air space area (mm^2^) was measured at E17.5 and PN21 with Kodak MI image system. Value are mean ± SE. *P* < 0.001 by one way Anova and Student’s *t*-test

We examined the lungs for any gross morphological abnormalities. Samples from PN21 mice revealed that the lung size of bi-transgenic mice is 40 % larger than the control littermates (Fig. 4a and b). However, the size is not significantly different in the lungs collected from E17.5 or PN0 among the bi-transgenic, single transgenic, and wild type mice (data not shown). Similarly, histological analysis of the pulmonary structure at E17.5 shows no defect in lung morphology (Fig. 4c and d). In contrast, the histopathological data revealed that the alveolar space in the bi-transgenic mice were significantly larger than those in the single transgenic or wild-type littermates at PN21 (Fig. 4e). After quantification with Kodak MI SE software and statistical analysis, the average alveolar space of the bi-transgenic mice is five times larger than control (Fig 4f). This observation indicates that ectopic expression of INSM1 disrupts alveolar septation that causes air space enlargement. It is likely that the INSM1 expression interrupts the last alveolar stage development in the bi-transgenic animal lung.

### INSM1 suppresses cyclin D1 expression in lung development

**Fig. 5:**
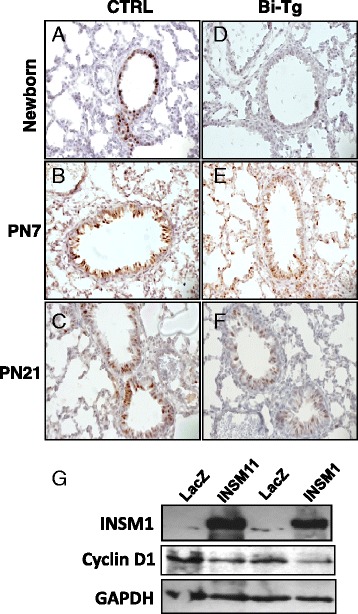
INSM1 ectopic expression suppresses cyclin D1 expression in bronchial epithelial cells. Immunohistochemical staining for cyclin D1, weaker and stronger positive signal were detected in the nuclei of bronchial epithelial cells in bi-transgenic mice (**d**-**f**) as compared to the control (**a**-**c**) on PN0, PN7, and PN21. Original magnification is 400X. **g** A human bronchial epithelial cell line, BEAS-2B, was infected with Ad-INSM1 or Ad-LacZ for 24 h and cultured in DMEM medium with 10 % fetal bovine serum for 14 or 24 h. The cell lysate was measured by Western blot analysis for INSM1 and cyclin D1 using housekeeping protein GAPDH as an internal control. Similar results were observed from three separated experiments

Immunohistochemical staining of control or bi-transgenic lung for cyclin D1 revealed that bi-transgenic lung has a weaker signal in the nuclei of bronchial epithelial cells and respiratory bronchiole epithelial cells (Fig. 5a-f). The results indicate that over-expression of INSM1 reduces cyclin D1 expression in club cells and CCSP-promoter active bronchial epithelial cells suggesting the reduction of cyclin D1 expression could cause cell cycle arrest and decreased cell proliferation.

### Ectopic expression of INSM1 suppresses cyclin D1 and induces cell cycle arrest in normal human bronchial epithelial cells, BEAS-2B

**Fig. 6:**
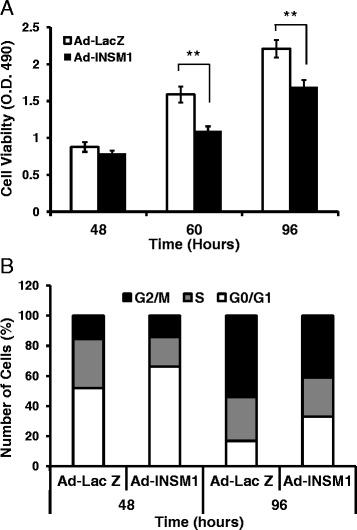
INSM1 ectopic expression retards cell growth and induces cell cycle arrest in bronchial epithelial cells, BEAS-2B. **a** BEAS-2B cells were infected with Ad-INSM1 or Ad-LacZ in serum-free medium for 24 h and then cultured in DMEM medium with 10 % fetal bovine serum for 48, 60 and 96 h. Cell viabilities were analyzed by MTS assay. **b** Cells were infected with Ad-INSM1 or Ad-LacZ for 48 and 96 h and stained with propidium iodium. DNA content was measured by FACS. Value are mean ± SE. *P* < 0.01 by Student’s *t*-test. ** indicate *P* < 0.01

Down regulation of cyclin D1 causes cell cycle arrest and interferes with bronchiolar epithelial cell proliferation. We used Ad-INSM1 or Ad-LacZ to infect a normal bronchial epithelial cell line, BEAS-2B. The results showed that over-expression of INSM1 caused the decrement of cyclin D1 (Fig. 5g). In vitro data of INSM1 suppressing cyclin D1 expression is consistent with the observation in our bi-transgenic mouse model where reduced cyclin D1 expression was seen in bronchial epithelial cells and club cells. To further support our hypothesis on the functional effects of INSM1 on bronchial epithelial cell growth, we over-expressed INSM1 in BEAS cells for 24 h in serum-free medium followed by serum stimulation at various time points and analyzed the cell growth with a MTS assay and flow cytometric analysis with propidium iodide staining. The MTS assay showed that INSM1 caused cell death starting at 48 h, as compared to the vehicle treated group. A statistically significant decrement of cell viability was found at 60 h (O.D_490_ = 1.091 v.s 1.58) and 96 h (O.D_490_ = 1.61 v.s 2.21) (Fig. 6a). The Ad-INSM1 infected cells showed reduced cell proliferation, as 32.86 % of the total population was found in the G_0_/G_1_ region as compared to 16.9 % of control Ad-LacZ infected cells (Fig. 6b). In addition, the G_2_/M population of cells in the Ad-INSM1 infected sample decreased to 40.98 % as compared to the Ad-LacZ control group, 54.18 %. This result suggests that over-expression of INSM1 caused cell cycle arrest at the G_0_/G_1_ phase probably through the reduction of cyclin D1 expression.

## Discussion

We initiated our study to dissect the functional effect of INSM1 transcription factor in normal lung development since INSM1 is a sensitive and specific NE lung cancer marker [7]. Club cells were found in close association with NEBs, where PNECs are derived from common multi-potent stem cells in the airway epithelium which is highly related to the non-ciliated secretory club cells. During lung development and injury, PNECs transiently express CCSP which is a major product of the non-ciliated progenitor cells for airway epithelium [3, 10]. Therefore, we chose CCSP-promoter driven ectopic expression of INSM1 as a model to reveal its functional effect during lung development. There are multiple genetically engineered mouse models for NE carcinomas of the lung [11]. The original *Rb/p53* double knockout [12] or triple knockout (double knockout model plus loss of *p130*) [13] or loss of *Pten* [14, 15] models resulted in multiple pulmonary tumors arising mainly from the central large bronchi with foci of in situ carcinoma and NE cell hyperplasia. An additional model with constitutive co-expression of SV40 large T antigen and human achaete-scute homolog-1 (hASH-1) generated adenocarcinomas with focal NE differentiation [16]. Solely ectopic expression of INSM1 in bronchial epithelial cell did not alter the lung epithelial cell toward NE differentiation. Whether the precursors were not properly targeted or ectopic expression of INSM1 is insufficient to induce NE differentiation and/or transformation is not known. The latter is more likely since constitutive expression of human achaete-scute homolog-1 (hASH-1) in combination with simian virus large T-antigen under the club cell CCSP-promoter resulted in adenocarcinomas with focal NE differentiation [16]. The expression of Mash1 and Insm1 are closely associated with NE differentiation [17].

This study revealed that INSM1 did not induce NE precursor differentiation instead it resulted in alveolar space enlargement and bronchial epithelial cell cycle arrest through down regulation of cyclin D1. One prominent function of club cells is to restore and renew the bronchiolar epithelial cells. Alveolar formation occurs at the final stage of lung development as the process begins at embryonic day 21 and continues entirely as a postnatal event in mice. Alveologenesis involves the septation of alveolar saccules into mature alveoli, which increases surface area and enhances the oxygen exchange capacity of the lung [18]. Cuboidal respiratory epithelial cell differentiation and proliferation play important roles in the septation process. Disruption of the process leads to alveolar hypoplasia that is characteristic in enlarged and simplified alveoli [19–21]. Down regulation of cyclin D1 will cause cell cycle arrest and decrease cell proliferation. Sequentially, less bronchiolar epithelial cells in the INSM1 over-expressed lung interrupt the repair and regeneration of the new bronchiolar epithelium and cause the air sac enlargement. Altered cyclin and Cdk expression consistent with G1 or G2 arrest has been reported in epithelial cells in the premature baboon model of BronchoPulmonary Dysplasia (BPD), a chronic lung disease that occurs in the premature infants and is characterized by impaired alveologenesis [22]. This result is consistent with our previous study that INSM1 induces non-NE cell cycle arrest by blocking the cyclin D1 and CDK4 interaction [23].

## Conclusion

A NE lung tumor marker was conditionally induced to express during lung development. The bi-transgenic animal model revealed that ectopic expression of INSM1 under the CCSP-promoter resulted in impairment of alveologenesis by increasing the air sac and causes alveolar hypoplasia. The defect is potentially caused by the reduction of cyclin D1 and cell cycle arrest during new bronchiolar epithelium regeneration.

### Ethics approval and consent to participate

Human small cell lung carcinoma tissue array (LC802b) was used in this study. Each specimen collected from any clinic was consented to by both hospital and individual. Discrete legal consent form was obtained and the rights to hold research uses for any purpose or further commercialized uses were waived (US Biomax, Rockville, MD). An IRB exemption (#8885) was obtained from Louisiana State University Health Sciences Center, New Orleans.

Animals were maintained in a pathogen-free vivarium in filtered cages according to the protocol approved by Institutional Animal Care and Use Committee from the Research Institute for Children, Children’s Hospital in New Orleans.

### Consent for publication

Not applicable.

### Availability of data and materials

Data and materials will be available for public upon request.

## Abbreviations

CCSP: club cell secretory protein
Dox: doxycycline
hASH-1: human achaete-scute homolog-1
INSM1: insulinoma-associated 1
NE: neuroendocrine
NEBs: neuro-epithelial bodies
PNECs: pulmonary neuroendocrine cells
SCLC: small cell lung cancer
